# 

**Published:** 1875-10

**Authors:** 


CONTENTS: PAGE

Dr. Hall's Picture, ........ 296
Hall's Journal for 1876, ...... 296
Pomarius, .... ........... .. 296
A Blessing or a Curse, .... 297
A Growing Vice, ...... . ...298
Long Dresses, ..... ......... 299
Habit, ..................... 300
A Word to Wives, ....... 300
Our Summer at Maplewood, Chap. IX. In the Orchard, 301
Protecting the Lungs, ...... 311
Saratoga in Winter, ..... 311
Drs. Hall & Gibbs, .........312
A Word for Women, ...... 312
The Hours of Sleep, ....... 313
Palate & Stomach, ......... 314

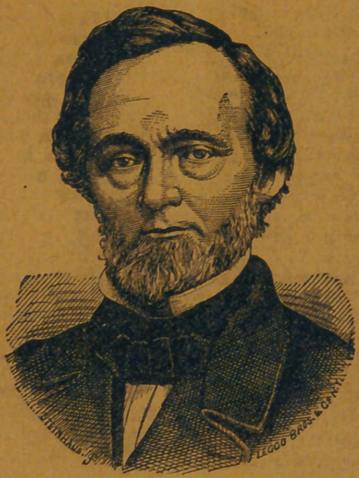

CONTENTS: PAGE

Secret of Good Looks, ..... 315
Health & Exercise, ....... 316
A Daily Need, ............. 317
Solid Leaven, ... .........318
A Revolution, .......... ... 319
A Great Industry, .........320
The Waves Defied, .. ... .321
Comeliness and Comfort, ....322
A Saving Salt, ............ 323
Mightier than the Pen, .... 324
Health and Comfort, ... ... 325
A Thing of Beauty, .....326
A Genuine Convenience, ....327
Dry Cold for Piles, .......328
The Marvelous Gum, ...... 328
Choice Wheat Product......330

